# Mapping secondary data gaps for social simulation modelling: A case study of the journeys of Syrian asylum seekers to Europe

**DOI:** 10.12688/openreseurope.15583.2

**Published:** 2024-10-07

**Authors:** Sarah Nurse, Martin Hinsch, Jakub Bijak

**Affiliations:** 1 University of Southampton, Southampton, SO17 1BJ, UK; 2 University of Glasgow, Glasgow, G12 8RZ, UK

**Keywords:** Agent-based modelling, Asylum migration, Data quality, Empirical evidence, Knowledge gaps, Modelling process, Secondary data, Syrian migration

## Abstract

Simulation models of social processes may require data that are not readily available, have low accuracy, are incomplete or biased. The paper presents a formal process for collating, assessing, selecting, and using secondary data as part of creating, validating, and documenting an agent-based simulation model of a complex social process, in this case, asylum seekers’ journeys to Europe. The process starts by creating an inventory of data sources, and the associated metadata, followed by assessing different aspects of data quality according to pre-defined criteria. As a result, based on the typology of available data, we are able to produce a thematic map of the area under study, and assess the uncertainty of key data sources, at least qualitatively. We illustrate the process by looking at the data on Syrian migration to Europe in 2011–21.

In parallel, successive stages of the development of a simulation model allow for identifying key types of information which are needed as input into empirically grounded modelling analysis. Juxtaposing the available evidence and model requirements allows for identifying knowledge gaps that need filling, preferably by collecting additional primary data, or, failing that, by carrying out a sensitivity analysis for the assumptions made. By doing so, we offer a way of formalising the data collection process in the context of model-building endeavours, while allowing the modelling to be predominantly question-driven rather than purely data-driven. The paper concludes with recommendations with respect to data and evidence, both for modellers, as well as model users in practice-oriented applications.

## Introduction

The aim of this brief report is to propose and reflect on an approach for collating, assessing, and selecting appropriate secondary data for use in an agent-based simulation model of a complex social process. The discussion is illustrated by a case study related to creating, validating, and documenting a model of migration route formation. Our intended contribution is to propose a template for critical assessment of secondary social data and for identifying key knowledge gaps, while remaining honest about the overall uncertainty of social simulation models, and the particular role of data uncertainty in it.

The work is motivated by the need to provide more realistic and reliable input to meet the ever-increasing policy demand for a better understanding of the patterns and drivers of migration. Our particular focus is on the rapidly-evolving asylum flows, where the relevant policies can be aimed at improving the preparedness of the transit and destination countries ^1^. The discussion is illustrated with an example of modelling Syrian asylum asylum seekers travelling to Europe in the 2010s, with the modelling process itself presented in more detail in Bijak *et al.* (2021). At the same time, in social simulation studies, data quality aspects rarely receive the attention they deserve.

This paper is structured as follows. First, in the Methods section, we discuss the interplay and tensions between the supply of available secondary data and the demand for such information to meet the specific needs and research objectives of social simulation models. We focus here particularly on the ways to identify the key information gaps. Subsequently, in the Results section, we present an example of data for a simulation model of migration route formation, based on the journeys made by Syrian asylum seekers travelling to Europe in the 2010s. The final section concludes with a discussion of key remaining challenges, possible solutions, and practical recommendations for model builders and users.

## Methods: Assessing data supply and demand

In empirically-grounded social modelling, a systematic review of the knowledge base typically begins with an assessment of the available secondary information on the topic in question. The foundations for that can be laid by formally creating a **comprehensive inventory of data supply**, as complete as possible, which aims to assemble key information and meta-information about the different types of data available, the key themes they refer to, and sources they come from. Such an inventory should ideally include meta-information about the classification of data according to a range of characteristics, such as whether the data source is qualitative or quantitative, is it a survey, census, register, observation, or interview, whether the level of aggregation is micro (individuals) or macro (groups), and so on. This meta-information can be either taken from the sources of the data themselves or imputed by analysts.

In the example concerning Syrian asylum seekers’ journeys to Europe in 2011–21, we have considered all publicly available datasets that could be freely accessible online and could potentially provide useful input into the simulation modelling of cross-Mediterranean migration routes. In particular, for data on migration flows and the context in which they occur, we have included a range of databases, online registers, provided by international and national organisations, such as UNHCR, Eurostat, Frontex, or national statistical offices, as well as those resulting from individual research studies, purposefully searched via Google Scholar. The meta-data collection took place in the summer of 2019, and was updated in the autumn of 2021, initially yielding 28 directly-relevant sources (after update increasing to 32), as well as 20 auxiliary ones, listed in Bijak *et al.* (2021).

The analysis of the limitations of the datasets, including of any biases, or other sources of uncertainty and errors, formed an inherent part of the next steps in evaluating the supply of available data. These steps consisted in establishing **a set of quality assessment criteria** suitable for the problem at hand. As data quality is a multidimensional construct, with different aspects having varying importance for different users and application areas, the assessment needs to be multidimensional, too. A simple and intuitive quality assessment scheme can use a variation of a traffic-lights approach (green for good data, amber for data with some problems, and red for poor data) to assess each quality dimension according to professional judgement (for related examples, see GAO, 2006, or Vogel and Kovacheva, 2008, and for a recent overview of issues with migration statistics generally, see Kraler and Reichel, 2022). ^1^

More broadly, international guidelines for data quality assessment in the context of the production of official statistics can be found e.g. in the documents of the United Nations Statistical Division (2018) and Eurostat (2019). For data quality, the latter guidance proposes to use five quality criteria (relevance; accuracy and reliability; timeliness and punctuality; coherence and comparability; and accessibility), while the former treats timeliness and punctuality separately, and additionally includes interpretability. However, these are generic data quality criteria, not specifically focused on modelling applications. The United Nations Statistical Division (2018) also explicitly notes that there are important trade-offs between different quality dimensions, the most notable of which is the tension between timeliness and accuracy – the more rapid the data production, the less space for thorough quality assurance.

In the example of data for modelling Syrian asylum journeys, we used a **five-point traffic-lights scale** inspired by the traffic-lights approach, with green-amber and amber-red as interim points. We use one threshold criterion, to what extent the data may be suitable for the problem at hand, followed by six specific criteria. Three of them concern the data generation process as such (its timeliness, the level of data disaggregation, as well as population coverage and adherence to definitions), two criteria refer to the level of trust in data (trustworthiness of the source and transparency of documentation), and one either to completeness (for register-based sources) or sample design (for surveys and other sample-based studies). Finally, we also provide a summary score averaging across the available quality dimensions – with individual scores treated numerically (from one for red to five for green), the summary score is an equally-weighted average across all the individual criteria, rounded to the nearest integer ( Bijak *et al*., 2021).

An initial stages of assembling a data inventory should also include a comprehensive documentation of all sources and classification decisions, as well as rationale for data assessment. The **analysis of metadata and data quality** **dimensions** can already shed light on the real extent of data supply potentially usable for modelling purposes. A summary of themes covered by different types of available data, filtered by those with a positive assessment of the relevant quality aspects, determines the actual, rather than potential data supply for the problem at hand. For example, by limiting the analysis to the theme of migrant journeys and considering only the sources falling between the green and amber categories overall, with a green rating for transparency and trustworthiness, already considerably limits the number of sources that can be used in the subsequent analysis. This exercise also helps illuminate the data gaps that would ideally need to be covered to meet the needs of modelling, if it is to be empirically grounded.

The other side of the process is related to the assessment of the **demand for secondary data** to be used in a simulation model. There are two main aspects of this. First, an analysis of data needs, initially at the conceptual level, can provide a rough idea what information would be ideally required for the model to have full empirical basis. Clearly, it is not possible to expect data on all aspects of the model, but this exercise already gives the first approximation about the areas, in which empirical grounding would be ideally needed. It also helps reflect on how the data can be operationalised—qualitatively or quantitatively, and through which variables—and how they can be used in the modelling process—whether to calibrate model parameters or other inputs, or to externally validate model outputs.

Once the initial assessment and modelling has taken place, the second step in assessing the demand for data can consist of an iterative process for **identifying the data gaps** that can be filled. From the point of view of the modelling process, the ultimate aim is either filling these gaps with secondary data, if available, or through a dedicated collection of primary data on a specific topic. The analytical mechanism which allows this involves *sensitivity analysis*, especially in the statistical sense, which identifies the model inputs that contribute the most to the model results ( Oakley & O’Hagan, 2004). Such inputs are primary targets for additional data collection. This process can be aided by formal modelling of the *provenance* (origins) of different data sources used in a model, and the relationships between the data and different modules and aspects of the model, identifying the data sources, on which many other elements of the modelled social reality are dependent ( Reinhardt *et al.*, 2023).

The identification of data gaps followed by collection of additional information can **proceed in an iterative manner** to reduce the gap between the demand for data and the available supply, while expanding the latter through addition of new, dedicated datasets. In the end, however, there are always some models’ aspects which cannot be calibrated to data, and some free parameters, for which empirical grounding is not possible. They are the key sources of residual uncertainty, which is an important indicator of model performance in its own right, and thus merits a separate analysis ( Bijak *et al.*, 2021).

## Results: Data on Syrian asylum seekers and refugees

In the presented example, we aim to study a migration process modelled on asylum flows from Syria to Europe during the 2010s. We use an agent-based approach, simulating individual migrants (agents), their journeys and decisions. The decisions are based on a range of factors known to the agents, but are made under uncertainty, resulting from incomplete information about the world, as well as knowledge and decisions of other agents (for details, see Hinsch & Bijak, 2023). The creation of the data inventory therefore had to encompass both individual-level data on Syrian asylum seekers and factors affecting their decisions, as well as macro-level information about the variables that could enter into the modelling process, either as input to decisions, or as a way of calibrating the model results. The inventory, covering 32 entries and including metadata for individual data sources, as well as their quality assessment, is available in Nurse and Bijak (2023) and in a searchable form from Nurse and Bijak (2021).

**Figure 1.**
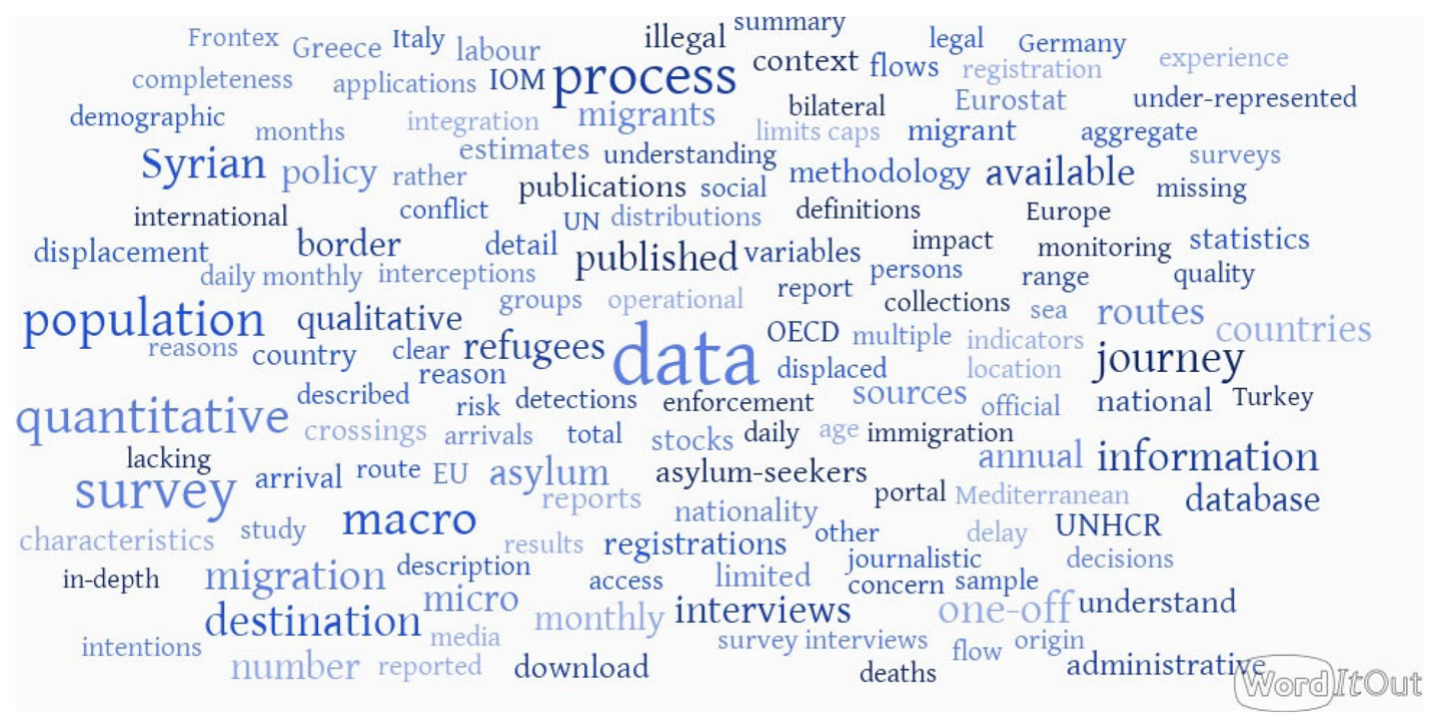
Textual description of the Syrian migration data inventory in a word cloud. Source: Nurse and Bijak (2021), created with WordItOut.com.

The data included in the inventory include both quantitative and qualitative sources that either pertain to the process of migrant journeys or the relevant context in which the journeys took place. The data could relate to individuals (micro-level), or be available at the aggregate, population levels (macro). **The inventory is purpose-specific** and covers only those sources that were publicly available at the time and that were deemed potentially relevant for modelling purposes. For this reason, the inventory may not cover those sources of data on Syrian migration that were either unavailable for research purposes or were found to be unrelated to the modelling objectives. What has been included, typically clustered around four main themes: data on routes and journeys as such, including on migrant decisions; data on the migrant populations, typically in destination countries; data on availability of information during journeys; and key data on relevant policies. The thematic map of the case study data is presented in Figure 1, displaying a word cloud created from the descriptions of all sources included in the inventory. The map is intended as a broad qualitative summary of the content of the inventory. This high-level exploratory overview points out to several key themes emerging from the data description: good availability of the population-level (macro) quantitative data on migration processes, but also the presence of at least some surveys and other micro-level data focused on journeys, including qualitative sources (interviews).

**Figure 2.**
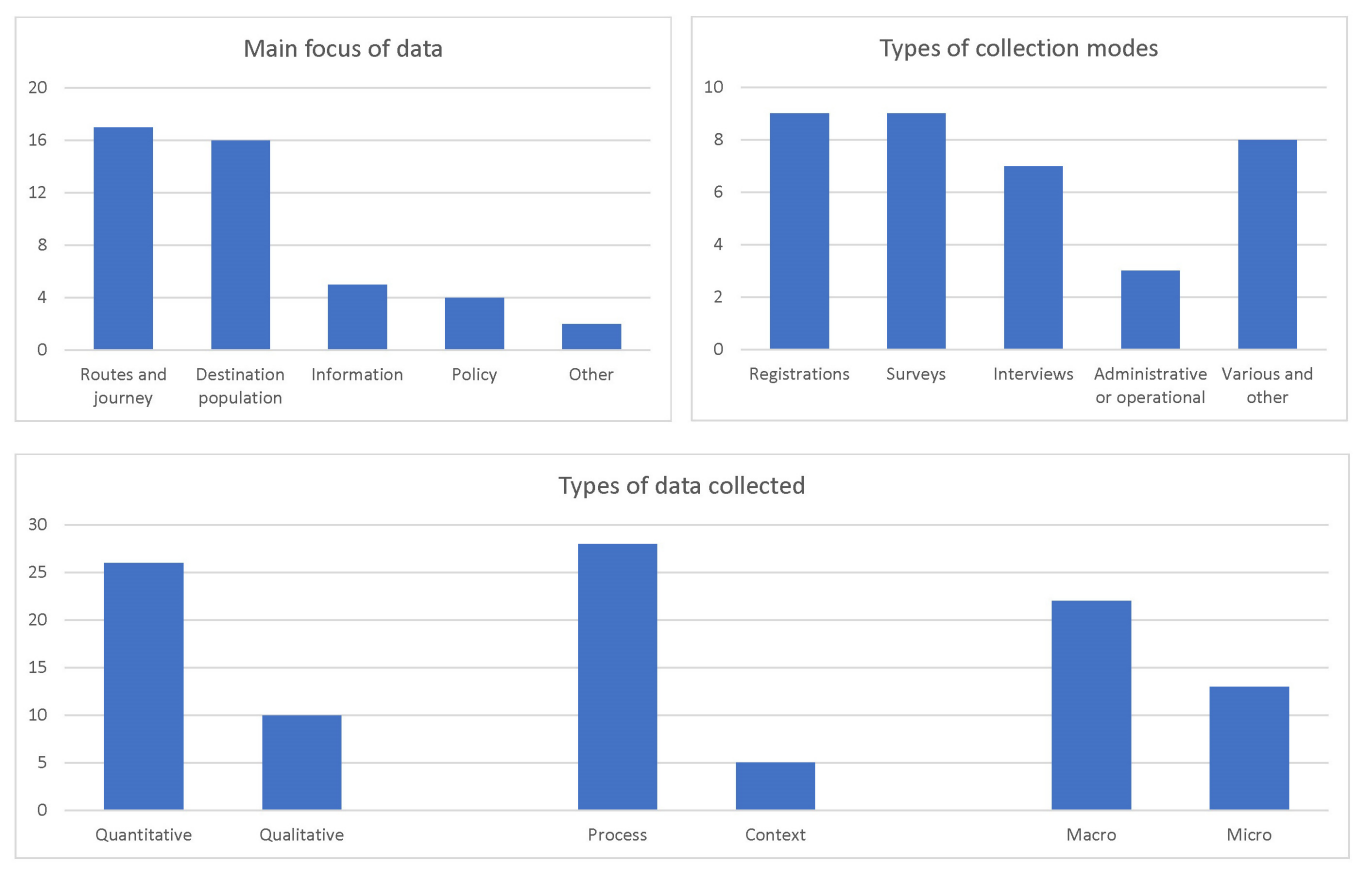
Focus, data types, and collection modes in the Syrian migration data inventory (numbers of sources). Source: Nurse and Bijak (2021).

A more detailed **summary of the inventory** is offered in Figure 2, which shows the frequencies (counts) of data sources included in the inventory. It is clear that the dominant groups of data sources covered migrant routes and journeys, as well as migrant populations in the destination countries. The data would typically come from either registers or surveys, routine or bespoke, closely followed by interviews with individual migrants. Most data included in the inventory are quantitative, related to migration process as such, and available at the aggregate level, although with a visible minority of qualitative, contextual and micro-level data as well.

**Figure 3.**
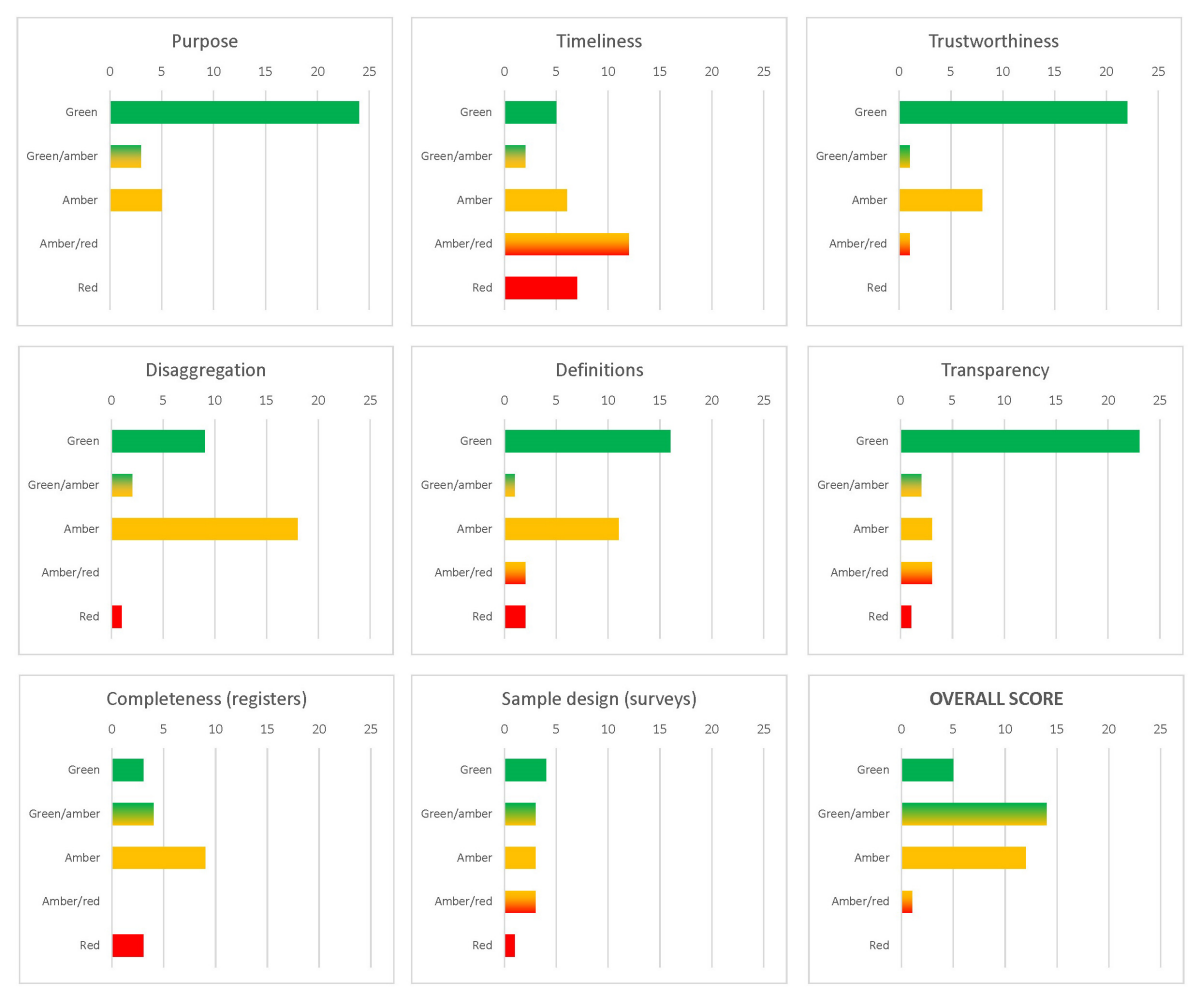
Quality dimensions of data sources included in the Syrian migration data inventory (numbers of sources). Source: Nurse and Bijak (2021).

The quality assessment for the sources included in the inventory is summarised in Figure 3. For all individual quality criteria, the expert assessment was carried out within the research team, and included an initial evaluation followed by independent moderation of the scores through a deliberative discussion and thought exchange. By definition of the inventory, all sources included had to be at least partially relevant for the modelling purposes. A clear majority of sources have also scored highly on the transparency and trustworthiness dimensions of the evaluation, as well as, although to a slightly smaller extent, on adherence to the established concepts and definitions. The quality assessment for source-specific criteria (sample design for surveys and completeness for registers) was mixed, with various sources located in different places along the five-point scale. The availability and quality of disaggregated data—for example by age or geographic detail—was typically average. The only more problematic dimension was related to timeliness: many data sources were one-off, resulting from specific projects rather than established collection efforts.

The overall quality rating, based on average scores summarising all quality dimensions with equal weights, indicated that the **available data supply is potentially useful for modelling**, especially after some corrections and adjustments. In addition to assessing the usefulness of available data for modelling, the quality dimensions also directly indicated the existing data gaps: what is potentially missing in the current body of evidence, in this case about Syrian asylum journeys. That timeliness was found particularly problematic points to an important data gap that could be filled through a dedicated asylum monitoring system, while issues with sample design or register completeness suggest the need for a methodological reflection on data collection mechanisms.

From the demand side, both the conceptual analysis of the model, as well as the formal statistical sensitivity analysis gave unequivocal results, identifying *information* as one of the primary factors shaping migrant journeys ( Bijak *et al.*, 2021; Hinsch & Bijak, 2023). At the same time, **information remains one of the key data gaps** and sources of model uncertainty: empirical data about information use by migrants and exchange between migrants are scarce. Usable micro-level data on information are limited to a single source: a comprehensive, albeit just one-off German survey *Flucht 2.0* ( Emmer *et al.*, 2016). In addition, modelling made use of aggregate estimates of the numbers of arrivals, border apprehensions, missing migrants and fatalities during journeys, originating from international organisations, such as UNHCR, International Organization for Migration, or Frontex (see Bijak *et al.*, 2021). In our models, the micro-level survey data ended up informing the assumptions about behavioural rules driving the agents’ behaviour, while the macro-level estimates were used for calibrating the model outputs. To correct for identified data quality issues, especially the biases (underestimation), the aggregate numbers had to be transformed into relative measures and annual rates of change.

Including empirical data enabled some reduction in the model uncertainty ( Bijak *et al.*, 2021). Still, bridging the data gap further would require additional collection of dedicated data, which in the example of modelling Syrian asylum seekers’ journeys has been attempted in follow-up work, both qualitative and quantitative ( Belabbas *et al.*, 2022; Bijak *et al.*, 2023), and verifying the remaining modelling assumptions through an extensive sensitivity analysis. In future work, the model construction can be also revisited, in order to provide a closer match to the available empirical basis, and **the process can be iterated, for as long as there are any information gains** and reduction in the model uncertainty.

## Discussion and conclusions

**Figure 4.**
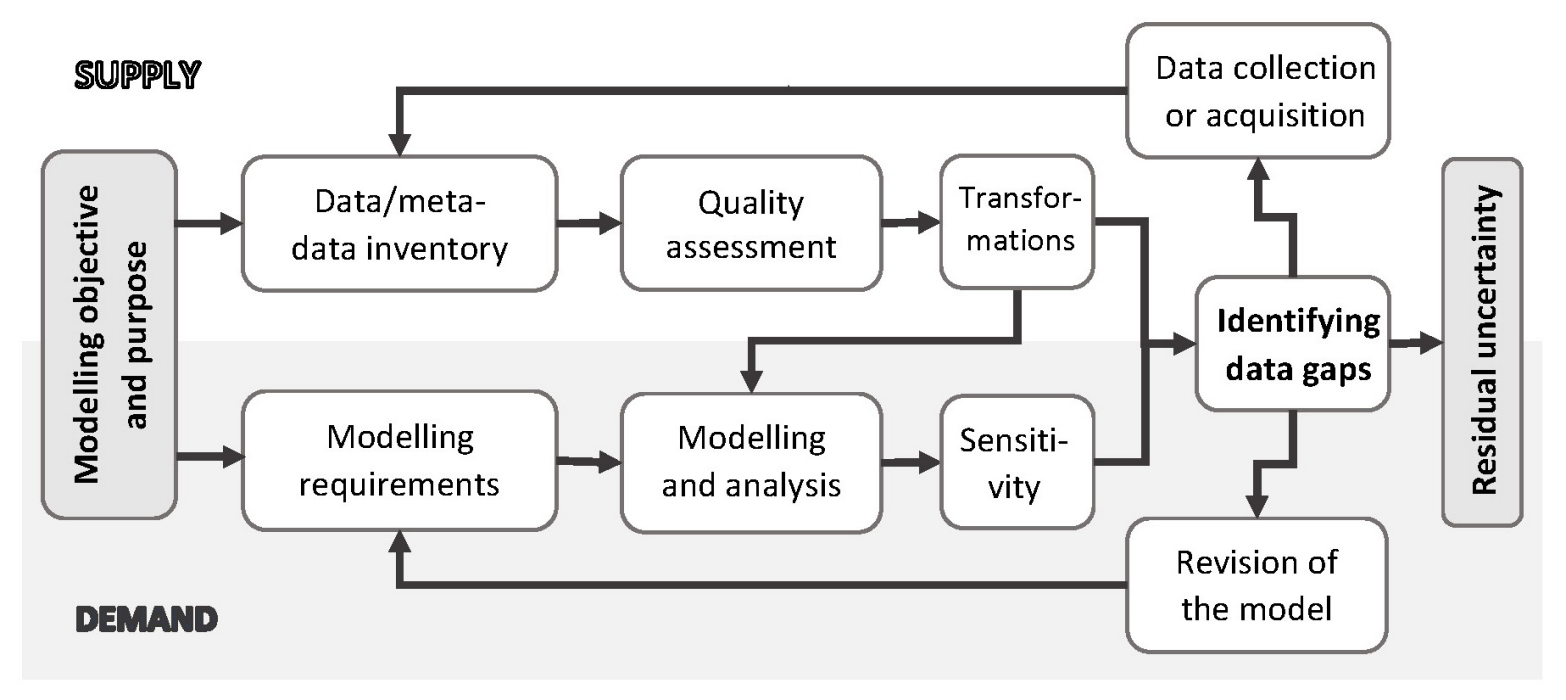
Iterative process for assembling and evaluating of secondary data for social simulation modelling. Source: Own elaboration.

In this note, we have described a process we propose for collating, assessing, selecting, and using secondary data for social simulation modelling purposes. The process, summarised in Figure 4, aims to reconcile the demand for data from the modelling side, with the available supply of reasonable-quality data that can be used to inform the model inputs or calibrate the outputs. The process is model specific, in that it always needs to begin by defining the needs defined by the particular modelling questions, with the data evaluated not only in their own right, but also with respect how well they serve the modelling objectives. The process then involves creating a dedicated data inventory, carrying out a quality assessment of the data, and analysis of data gaps both directly, from the quality assessment, as well as indirectly, through the lens of the modelling results. The identified gaps allow focusing on specific areas of further primary data collection and sensitivity analysis.

The example of our modelling exercise, discussed in the previous section, can illustrate the workings of this process. Having defined the purpose and objective of data collection (to inform the modelling Syrian asylum journeys to Europe), enabled to set out the initial modelling requirements in terms of data on migrant decisions, information, and macro-level processes. At the same time, from the available data, collated and assessed for quality by following the template discussed above, we were able to identify a few sources that could be potentially useful for modelling. The macro-data, after transforming, were used for model calibration, and the *Flight 2.0* survey data provided important insights into the role of information in migration decisions. These data sources were used directly in the modelling process, but also leaving important gaps, reflected through residual model uncertainty. The sensitivity analysis showed information to be one of key gaps, which suggests a potentially fruitful area for further data collection in the future.

Any limitations notwithstanding, our findings point out to several practical implications for social simulation modellers with respect to the gathering of data and evidence for modelling. First, as summarised in Figure 4, the process of modelling enquiry needs to be driven by a specific research objective and purpose, and clearly acknowledge the limitations and uncertainty of empirical data. Second, data quality and importance in a given model needs to be formally analysed, both conceptually and through statistical means. Third, to facilitate that, data need to be thoroughly documented, for example by using provenance standards ( Reinhardt *et al.*, 2023).

Finally, there are important trade-offs between the degree of empirical grounding and the level of detail of mechanistic realism of social simulation models. The more complicated the models, the fewer model elements can have reliable empirical basis, and the greater need for more disaggregated data – even though the aggregates are still likely to remain crucial, if only for calibration. At the same time, any users of social simulation models, especially in policy and practice-related applications, need to be made explicitly aware of data-related limitations. The need to take data uncertainty formally and openly into account when modelling is paramount both for transparency and with respect to the limits of scientific knowledge. This is especially important for such complex and politically charged topics as migration.

As an important caveat, the presented quality assessment of data sources has been inevitably based on expert judgement and has been arrived at through a deliberative process within the research team. For future extensions, one possible way of making the assessment more robust, would be to give it more formal structure, for example through a Delphi survey amongst many experts. Another could involve adding more specific classification rules for every criterion, for example accompanied by decision trees where a certain set of answers to questions about data would imply the final quality rating. In our exercise, however, we have found the process presented in this paper to be sufficiently fine-tuned to be able to identify the potentially-useful data sources and point to their shortcomings, such as biases. This in turn enabled correcting these shortcomings through appropriate data transformations and by making realistic assumptions about the errors.

Given that our quality assessment criteria are generic, and align well with the professional standards in official statistics (e.g. United Nations Statistics Division, 2018; Eurostat, 2019), the proposed framework should be broadly transferrable to other contexts, possibly with only minor modifications. The purpose can, of course, extend beyond modelling, and in other situations various criteria may be given different weighting, but the broad principles remain the same. Still, one additional aspect which may become important especially in some areas of application is the responsible use, including – but not limited to – data protection and security ( IASC, 2023). It is easy to imagine contexts in which the analysis of especially individual-level data, if done carelessly or by malevolent actors, can result in harm to people or communities. In such situations, including additional criteria related to robustness of the data against possible misuses would be critical to safeguard the key ethical imperative of any well-intentioned social analysis: do no harm.

## Ethics and consent

Ethical approval and consent were not required.

## Data Availability

Zenodo: Syrian Migration to Europe, 2011–21: Data Inventory. https://doi.org/10.5281/zenodo.7586826 ( Nurse & Bijak, 2023). The project contains the following underlying data: Syrian_migration_2011–21_data_inventory.tsv Syrian_migration_2011–21_data_inventory.xlsx Data are available under the terms of the Creative Commons Attribution 4.0 International license (CC-BY 4.0). No primary data are associated with this article, as the analysis presented herein is based on a conceptual analysis of publicly-available metadata of various migration data sources, summarised in an online inventory https://baps-project.eu/inventory/data_inventory ( Nurse & Bijak, 2021). The description of the agent-based simulation model of migration route formation used in this paper is available via https://baps-project.eu/inventory/project_outputs and the model itself is discussed in more detail in Hinsch and Bijak (2023).
