# Supplementary material for: Mapping secondary data gaps for social simulation modelling: A case study of the journeys of Syrian asylum seekers to Europe
Source: Open Res Eur. 2024 Oct 7;3:216. Originally published 2023 Dec 8. [Version 2] doi: 10.12688/openreseurope.15583.2 (PMC10873545; doi:10.12688/openreseurope.15583.2)
Supplement: Peer review history

# Reviewer report: Reviewer response for version 2

**DOI:** 10.21956/openreseurope.20171.r44897

**Published:** 2024-10-14

**Authors:** Alejandra Rodriguez-Sánchez

**Affiliations:** 1 University of Potsdam, Potsdam, Germany

I have read the manuscript and author responses to my previous comments and I find the paper is now in a better form.

If applicable, is the statistical analysis and its interpretation appropriate?

Yes

Are all the source data and materials underlying the results available?

Yes

Is the study design appropriate and is the work technically sound?

Partly

Is the work clearly and accurately presented and does it engage with the current literature?

Yes

Are the conclusions drawn adequately supported by the results?

Yes

Are sufficient details of methods and analysis provided to allow replication by others?

Partly

Reviewer Expertise:

NA

I confirm that I have read this submission and believe that I have an appropriate level of expertise to confirm that it is of an acceptable scientific standard.

# Reviewer report: Reviewer response for version 1

**DOI:** 10.21956/openreseurope.16843.r37144

**Published:** 2024-02-16

**Authors:** Denis Kierans

**Affiliations:** 1 University of Oxford, Oxford, England, UK

The article outlines a carefully thought out approach to making sense of data that are of variable quality but carry high levels of political and operational significance. Of particular interest is the transparency the authors provide by reflecting on the types of data under review; the creation of categories that fall between the typical green, amber and red; the decision to prioritise high levels of trustworthiness and transparency among the various criteria. The article is clear about the limits of modelling in this area, which is also well received. For these reasons and more this succinct article is a fine contribution and will hopefully lead to further well-documented, sober reflections on data quality (etc.) in other areas of social science research.

If applicable, is the statistical analysis and its interpretation appropriate?

Yes

Are all the source data and materials underlying the results available?

Yes

Is the study design appropriate and is the work technically sound?

Yes

Is the work clearly and accurately presented and does it engage with the current literature?

Yes

Are the conclusions drawn adequately supported by the results?

Yes

Are sufficient details of methods and analysis provided to allow replication by others?

Yes

Reviewer Expertise:

Areas of research: migration, migration data, migration policy, integration.

I confirm that I have read this submission and believe that I have an appropriate level of expertise to confirm that it is of an acceptable scientific standard.

# Response

**Published:** 2024-09-26

**Authors:** Jakub Bijak

**Affiliations:** University of Southampton, UK

Thank you very much for your review and very kind words: we are delighted to hear you find the material useful!

# Reviewer report: Reviewer response for version 1

**DOI:** 10.21956/openreseurope.16843.r37139

**Published:** 2024-02-16

**Authors:** Alireza Jahani

**Affiliations:** 1 Brunel University London, London, England, UK

The paper provides a detailed and well-structured methodology for collating, assessing, and selecting secondary data for social simulation modelling, with a specific focus on Syrian asylum migration. The paper begins by outlining the need for more realistic and reliable data to meet the increasing demand for policy-oriented insights into migration patterns. It emphasizes the importance of assessing data quality and identifying knowledge gaps in the context of modelling endeavours. The structured approach involves creating a comprehensive inventory of data sources, assessing data quality using predefined criteria, and mapping the thematic areas to visualize data uncertainty.

 The methodology presented in the "Methods" section is clear and well-defined. The authors rightly highlight the multidimensional nature of data quality, covering aspects such as timeliness, level of aggregation, trustworthiness, and transparency. The use of a traffic-light approach and a summary score provides a practical and intuitive way to evaluate data quality. The inclusion of a case study on Syrian asylum migration enhances the paper's applicability and demonstrates the methodology in action.

 As an expert in the field that modelled the Syrian conflict forced migration before, I find the approach to be comprehensive, and the paper addresses a critical aspect often overlooked in social simulation studies—the quality and availability of secondary data. I remember we wanted to couple our model with telecommunication dataset as secondary data and faced a lot of challenges.

 In the "Results" section, I expected to see more details of the case study, with a focus on the data inventory, thematic mapping, and quality assessment of available data. However, I believe that the identification of data gaps and the iterative process of data collection are particularly valuable insights for researchers and practitioners alike.

 The paper concludes with a thoughtful discussion on the challenges, solutions, and recommendations for social simulation model builders and users. It emphasizes the importance of a question-driven rather than purely data-driven approach, acknowledging the inherent uncertainty in social simulation models. The iterative process outlined in the discussion offers a practical way to bridge data gaps and improve model reliability.

 Overall, the paper is a valuable contribution to the field of social simulation modelling. It provides a systematic and transparent approach to handling secondary data in modelling endeavours, making it accessible to both experts and non-experts in the field. While the paper is comprehensive, providing more illustrative examples or specific insights from the case study could further enhance its impact and clarity for a broader audience.

If applicable, is the statistical analysis and its interpretation appropriate?

Yes

Are all the source data and materials underlying the results available?

Yes

Is the study design appropriate and is the work technically sound?

Yes

Is the work clearly and accurately presented and does it engage with the current literature?

Yes

Are the conclusions drawn adequately supported by the results?

Yes

Are sufficient details of methods and analysis provided to allow replication by others?

Partly

Reviewer Expertise:

Forced Displacement Modelling and Simulation

I confirm that I have read this submission and believe that I have an appropriate level of expertise to confirm that it is of an acceptable scientific standard.

# Response

**Published:** 2024-09-26

**Authors:** Jakub Bijak

**Affiliations:** University of Southampton, UK

Thank you very much for your review and the endorsement of the paper. In the initial version, we were constrained by the limits of the Research Brief format, but we have now added some more details on the case study, with particular focus on the assessment process, mapping of the available data, and how the results of the exercise were subsequently used in the modelling. We hope this helps explain the process and our intentions behind it clearer.

# Reviewer report: Reviewer response for version 1

**DOI:** 10.21956/openreseurope.16843.r36789

**Published:** 2024-01-30

**Authors:** Edgar Scrase, Zaruhi Mkrtchyan

**Affiliations:** 1 United Nations High Commissioner for Refugees (UNHCR), Geneva, Switzerland; 2 Global Data Service, UNHCR, Copenhagen, Denmark

We welcome this research – systematizing the review of secondary data is useful, and relevant.  There are many research teams that could benefit from such a structured approach.  The approach is well presented and the structure and flow of the paper are good with a logical progression from presenting the framework and then unpacking it as a case study. The Syrian refugee example is relevant and serves as a practical example of how the theoretical model can be implemented and operationalized.

 The framework described to assess the quality of the data is useful.  We would welcome greater explanation of how the traffic light colours were assessed – i.e. are there a series of criteria in each quality dimension that lead to a general score?  Or is it an expert assessment of each dataset?  Additional detail on this would help readers to operationalize the approach in their own research.

 There is also no mention of statistical quality assurance frameworks such as the work by UN stats division ( https://unstats.un.org/unsd/unsystem/documents/UNSQAF-2018.pdf) and the European Statistic System ( https://ec.europa.eu/eurostat/documents/64157/4392716/ESS-QAF-V1-2final.pdf/bbf5970c-1adf-46c8-afc3-58ce177a0646).  These are well researched and describe a broader set of quality dimensions that are relevant to this research.  The UNSD SQAF in particular also describes the inherent compromises that need to be made between quality dimensions (i.e. prioritising timeliness over accuracy etc.) which could usefully be mentioned in this paper.  The broader set of quality dimensions could also help to provide a set of criteria that could be more broadly applicable to other research.  For example, greater mention could be made about using data responsibly.  It’s implicit in this research example as only public data is used, but in other research some of the qualitative individual data may contain personal data, for which the researchers and ultimately the data owners must take responsibility in assessing whether it is appropriate to publish such data or even to use it in the research.  This is well covered in this IASC paper on data responsibility ( https://interagencystandingcommittee.org/operational-response/iasc-operational-guidance-data-responsibility-humanitarian-action).

 A broader set of quality dimensions would also cover data availability / data gaps as well, which are mentioned in the results section.  I.e. for the quality dimensions to be truly useful they would ideally help to assess both the available data as well as what is potentially missing.

 In terms of terminology, we would suggest to avoid “asylum migration” as a term as it conflates two reasons for people moving.  Either the Syrians are seeking asylum in order to become refugees or they are migrating for other reasons.  We would suggest “Syrian refugees arriving in Europe” for the article.

 Thanks again for the opportunity to review, all work like that expressed in this paper, to operationalise quality improvements to data and data processing are very welcome and we would be interested to collaborate in future activities in this domain.

If applicable, is the statistical analysis and its interpretation appropriate?

Yes

Are all the source data and materials underlying the results available?

Yes

Is the study design appropriate and is the work technically sound?

Yes

Is the work clearly and accurately presented and does it engage with the current literature?

Yes

Are the conclusions drawn adequately supported by the results?

Yes

Are sufficient details of methods and analysis provided to allow replication by others?

Partly

Reviewer Expertise:

Forced displacement population statistics

We confirm that we have read this submission and believe that we have an appropriate level of expertise to confirm that it is of an acceptable scientific standard, however we have significant reservations, as outlined above.

# Response

**Published:** 2024-09-26

**Authors:** Jakub Bijak

**Affiliations:** University of Southampton, UK

Thank you for very careful reading of the paper and for very helpful comments.  In the first version, we opted for brevity, to meet the constraints of the Research Brief submission, but we agree that additional details would be helpful, including on the aggregation of quality scores, as pointed out also by the other reviewers. We hope the new additions in the Methods and Results sections are helpful, and we summarise the key changes below.

- Many thanks for the excellent references to the official statistics standards on data quality and the associated documentation (which we have now added in the Methods section), and to the point about responsible use of data, with which we fully concur, and are now mentioning in the Discussion and Conclusions. We allow ourselves to credit you for the idea, but agree wholeheartedly about the importance of the ethical reflection for data use, especially at the individual level.
- Thank you also for the suggestions of additional quality dimensions, which we have now mentioned in the Results and Discussion and Conclusions of the paper as the direct result of the application of the evaluation framework (as opposed to indirect, following the modelling results).
- As to the terminology, your point is well taken: even though contemporary migration scholarship would point to multiple motives for different types of mobility, including asylum, we agree that a more precise term would help. We have thus pivoted to "Syrian asylum seekers" throughout, as this is about people who have not (yet) been granted refugee status, although we also mention "refugees" in the context of the database, which may include data relevant to this population.

# Reviewer report: Reviewer response for version 1

**DOI:** 10.21956/openreseurope.16843.r37138

**Published:** 2024-01-24

**Authors:** Alejandra Rodriguez-Sánchez

**Affiliations:** 1 University of Potsdam, Potsdam, Germany

This paper presents an approach for collating assessing and selecting appropriate secondary data to build agent-based simulation models of migration. It is applied to the specific case of the Syrian asylum migration to Europ during the 2010s.

 Evaluating the information available for modelling analysis is a fundamental aspect of model development. My main take from the paper is on the importance of incorporating data critically in the building of an ABM for migration.

 However, here are some recommendations and questions that might improve the manuscript and the exposition of the method:

- The paper could make clearer how the discussion of the data quality led to decisions in the modeling part. This is left implicit, but readers could benefit from that.
-  Figure 1, a word cloud, is rather uninformative and includes terms that aren't relevant (e.g., rather, since, other, detail etc.). I am not sure if this was the starting point for doing something else on the basis of word frequency, like classifying the types of data sources.
-  The quality assessment of the data sources seems rather ad hoc. Here is perhaps where some notion of a standard could be applicable or at least determined. What is a high quality data source? What is a bad quality? Isn't a bad quality data source useful for modelling in the absence of any other information?
-  On a similar note, depending on the type of data, an evaluation of its quality will have to follow different criteria. How were criteria differently reconciled here? And more specifically, how did you arrive at the score in the table? Would it be possible to describe, at least for some case, how did you come to evaluate a data source as green-amber v. amber? In general, the quality of a data source should be evaluated on the basis of a given model (how useful is it to model that specific element of the phenomenon to be simulated). Is it something like the more detailed the model, the less useful aggregate data might be?
- If this piece is to serve as a template, as described by the authors, it should have such a structure. The diagram in figure 4 is, in this sense, quite illustrative, but I miss a reflection of each of the elements, perhaps in a separate paragraph, that walks the reader through an example of a data source and how it affected the modelling.
- What factors would we have to consider if we were interested in modeling other forms of migration or migration in other parts of the world? How specific or transferable are the different steps in this template?

All in all, authors are attempting to provide guidance on how to incorporate data into the building of ABMs, which would be a great contribution.

If applicable, is the statistical analysis and its interpretation appropriate?

Yes

Are all the source data and materials underlying the results available?

Yes

Is the study design appropriate and is the work technically sound?

Partly

Is the work clearly and accurately presented and does it engage with the current literature?

Yes

Are the conclusions drawn adequately supported by the results?

Yes

Are sufficient details of methods and analysis provided to allow replication by others?

Partly

Reviewer Expertise:

Migration, family demography, causal inference

I confirm that I have read this submission and believe that I have an appropriate level of expertise to confirm that it is of an acceptable scientific standard, however I have significant reservations, as outlined above.

# Response

**Published:** 2024-09-26

**Authors:** Jakub Bijak

**Affiliations:** University of Southampton, UK

Thank you for the careful reading of the paper and for your feedback. We have now, also in response to the other reviewers, expanded on the description of the process of incorporating the data quality assessment in the modelling, in the Results section, which we hope will help the readers see not only the details of the process, but also its end-point. Our responses to your specific comments are listed below:

- Regarding the word cloud map in Figure 1, it was meant to visualise the thematic map of the inventory, which we now state explicitly in the text. You are right that some terms may be common words, although would disagree about some specifics (e.g. to us ‘other’ carries extra meaning in that ‘other’ means ‘outside of pre-defined categories’). Still, we have cleaned the figure by removing some of the obviously irrelevant terms and have expanded the discussion, to include the exploratory character of this de facto qualitative analysis of the database content.
- As to the assessment of data sources, it is inevitably based on some dose of judgement, which does not necessary mean ad hoc, as it has been arrived through a deliberative process within the team – here, one possible way of making the assessment more robust, would be to give it more formal structure, for example through a Delphi survey amongst experts, or similar. In addition, We now also explain the process of aggregating the quality scores more clearly.
- Thank you also for the request to explain the aggregation of individual criteria clearer, which we have now attempted in the current version of the paper. You are right to point out that the assessment is model-specific, and we now make this point explicit. The point about aggregate data is more subtle, but we attempt spelling it out as well: in brief, the more detailed the model, the greater the need for disaggregated data, but higher-level aggregates are still likely to be useful for benchmarking the project output.
- The decision not to include specific examples was driven by the initial constraints of the Research Brief format, but in this version, besides adding a comment on the process by which the data assessment enters into the modelling, we have also provided cross-references to two example sources of data (UNHCR and Flucht 2.0), to illustrate it better.
- Finally, we have added a comment on transferability to the Discussion and conclusion part of the paper, with thanks for the suggestion – we agree it adds value to the argument.
